# Self-Assembled Recombinant Elastin and Globular Protein Vesicles with Tunable Properties for Diverse Applications

**DOI:** 10.1021/acs.accounts.3c00694

**Published:** 2024-04-16

**Authors:** Mikaela A. Gray, Mariela R. Rodriguez-Otero, Julie A. Champion

**Affiliations:** † School of Chemical and Biomolecular Engineering, Georgia Institute of Technology, 950 Atlantic Dr NW, Atlanta, Georgia 30332, United States; ‡ BioEngineering Program, Georgia Institute of Technology, 950 Atlantic Dr NW, Atlanta, Georgia 30332, United States

## Abstract

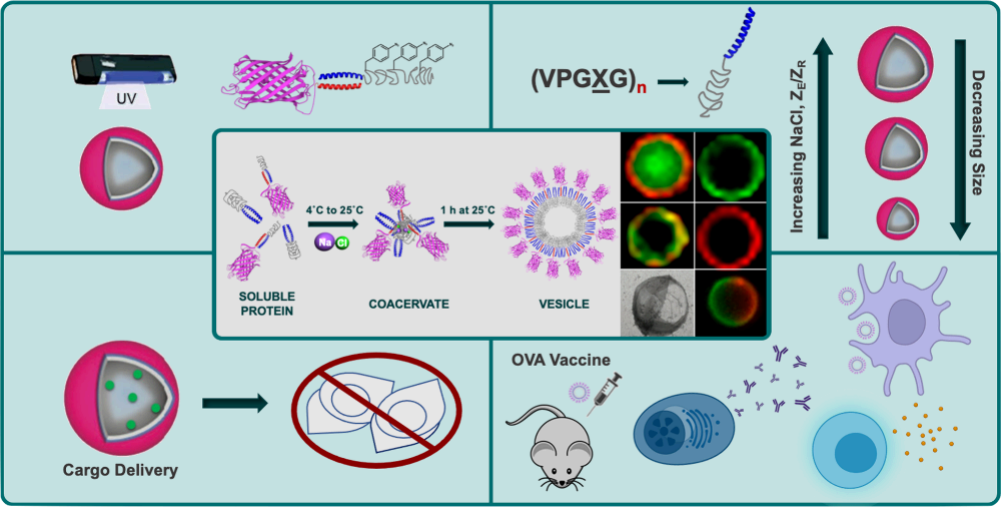

Vesicles are self-assembled structures comprised of a membrane-like exterior surrounding a hollow lumen with applications in drug delivery, artificial cells, and micro-bioreactors. Lipid or polymer vesicles are the most common and are made of lipids or polymers, respectively. They are highly useful structures for many applications but it can be challenging to decorate them with proteins or encapsulate proteins in them, owing to the use of organic solvent in their formation and the large size of proteins relative to lipid or polymer molecules. By utilization of recombinant fusion proteins to make vesicles, specific protein domains can be directly incorporated while also imparting tunability and stability. Protein vesicle assembly relies on the design and use of self-assembling amphiphilic proteins. A specific protein vesicle platform made in purely aqueous conditions of a globular, functional protein fused to a glutamate-rich leucine zipper (Z_E_) and a thermoresponsive elastin-like polypeptide (ELP) fused to an arginine-rich leucine zipper (Z_R_) is discussed here. The hydrophobic conformational change of the ELP above its transition temperature drives assembly, and strong Z_E_/Z_R_ binding enables incorporation of the desired functional protein. Mixing the soluble proteins on ice induces zipper binding, and then warming above the ELP transition temperature (*T*_t_) triggers the transition to and growth of protein-rich coacervates and, finally, reorganization of proteins into vesicles. Vesicle size is tunable based on salt concentration, rate of heating, protein concentration, size of the globular protein, molar ratio of the proteins, and the ELP sequence. Increasing the salt concentration decreases vesicle size by decreasing the *T*_t_, resulting in a shorter coacervation transition stage. Likewise, directly changing the heating rate also changes this time and increasing protein concentration increases coalescence. Increasing globular protein size decreases the size of the vesicle due to steric hindrance. By changing the ELP sequence, which consists of (VPGXG)_*n*_, through the guest residue (X) or number of repeats (*n*), *T*_t_ is changed, affecting size. Additionally, the chemical nature of X variation has endowed vesicles with stimuli responsiveness and stability at physiological conditions.

Protein vesicles have been used for biocatalysis, biomacromolecular drug delivery, and vaccine applications. Photo-cross-linkable vesicles were used to deliver small molecule cargo to cancer cells *in vitro* and antigen to immune cells *in vivo*. pH-responsive vesicles effectively delivered functional protein cargo, including cytochrome C, to the cytosol of cancer cells *in vitro*, using hydrophobic ion pairing to improve cargo distribution in the vesicles and release. The globular protein used to make the vesicles can be varied to achieve different functions. For example, enzyme vesicles exhibit biocatalysis, and antigen vesicles induce antibody and cellular immune responses after vaccination in mice. Collectively, the development and engineering of the protein vesicle platform has employed amphiphilic self-assembly strategies and rational protein engineering to control physical, chemical, and biological properties for biotechnology and nanomedicine applications.

## Key References

- JangY.; ChoiW. T.; HellerW. T.; KeZ.; WrightE. R.; ChampionJ. A.Engineering Globular Protein Vesicles through Tunable Self-Assembly of Recombinant Fusion Proteins. Small2017, 13 ( (36), ), 170039910.1002/smll.20170039928748658.^1^ Determined the driving forces and stages of protein vesicle self-assembly and demonstrated how assembly process conditions could be manipulated to control vesicle size and whether single or bilayered vesicles formed.
- DautelD. R.; ChampionJ. A.Protein Vesicles Self-Assembled from Functional Globular Proteins with Different Charge and Size. Biomacromolecules2021, 22 ( (1), ), 116–12532886493 10.1021/acs.biomac.0c00671.^2^ Modified globular protein size and charge to determine their influence on vesicle self-assembly, developed vesicle phase diagrams for salt concentration and protein composition, and demonstrated biocatalytic activity of enzyme vesicles.
- LiY.; DautelD. R.; GrayM. A.; McKennaM. E.; ChampionJ. A.Rational Design of Elastin-like Polypeptide Fusion Proteins to Tune Self-Assembly and Properties of Protein Vesicles. J. Mater. Chem. B2023, 11, 644337357544 10.1039/d3tb00200d.^3^ Utilized protein engineering to manipulate ELP guest residue hydrophobicity and length to control ELP transition temperature and reveal the effect of transition temperature on vesicle size and stability.

## 1 Introduction

Vesicles are compartments composed of hydrophilic and hydrophobic moieties forming a membrane-like structure. Liposomes, vesicles formed from lipids, are naturally abundant and used in pharmaceutical formulations, but exhibit limited stability.^4^ Polymersomes are vesicles composed of amphiphilic block copolymers and have been engineered to control permeability, size, shape, and stability.^5−10^ However, polymersomes have lower biofunctionality and biocompatibility than liposomes.^11^ To form tunable and biocompatible vesicles, recombinant proteins have been used. Hammer and co-workers engineered the self-assembly of oleosin protein vesicles, tuning them based on the fraction of hydrophilic and hydrophobic amino acids.^12^ Functional peptides can be incorporated, such as integrin binding ligand (RGD) or collagen like peptide, to impart targeting.^13^ More recently, Schreiber and co-workers designed amphiphilic elastin-like polypeptides (ELPs), which encapsulate a variety of cargos and self-assemble into bilayered vesicles ranging in size from 0.4 to 2 μm in diameter depending on assembly conditions.^14^ Additionally, Lecommandoux and co-workers designed lipid-grafted ELPs that allowed vesicle formation with controlled permeability.^15^ Moreover, Schiller and co-workers elucidated the self-assembly of tailored amphiphilic ELPs into supramolecular protein assemblies such as unilamellar vesicles, spherical coacervates, and twisted fiber bundles.^14^ In all examples, self-assembly of protein vesicles is facilitated by the packing of peptide amphiphiles and is characterized by an aqueous core and a membrane that is organized to shield hydrophobic domains from the aqueous phase. Vesicles made from proteins have tunable functionalization, biocompatibility, and biodegradability. This Account covers the fundamentals of self-assembly of protein vesicles made from globular proteins, leucine zippers, and ELPs made in the Champion Lab, and use of both process and protein engineering methods to modify vesicle size, morphology, and stability for applications including drug delivery, vaccines, and biocatalysis.

## 2 Recombinant Elastin and Globular Protein Vesicle Fundamentals

Protein vesicles self-assemble in an aqueous environment from recombinant protein amphiphiles with a temperature-induced phase transition. Two fusion proteins, globule-Z_E_ and Z_R_-ELP, are recombinantly expressed in *Escherichia coli* to use as vesicle building blocks.^16^ Globule stands for a functional, globular, folded protein, which in fundamental studies has been mCherry, a model fluorescent protein. mCherry is genetically fused to an acidic glutamate-rich leucine zipper (Z_E_). Z_R_-ELP contains ELP fused to a basic arginine-rich leucine zipper (Z_R_). ELPs are derived from human tropoelastin and are pentapeptide repeats of amino acids Val-Pro-Gly-Xaa-Gly (VPGXG, where X is a guest residue).^17^ ELPs exhibit lower critical solution temperature phase behavior as they are heated. ELPs have characteristic transition temperatures (*T*_t_), defined as the midpoint temperature of their reversible phase transition. ELPs are soluble well below *T*_t_ and upon warming undergo a hydrophobic conformational change as they approach and surpass *T*_t_. Warming induces phase separation of protein-rich liquid droplets, or coacervates, which are aggregates that form a dense viscoelastic phase.^17−20^ This conformational change reverses upon cooling.^20^*T*_t_ depends on guest residue, ELP length, salt concentration and type, and protein concentration.^21−24^

**Figure 1:**
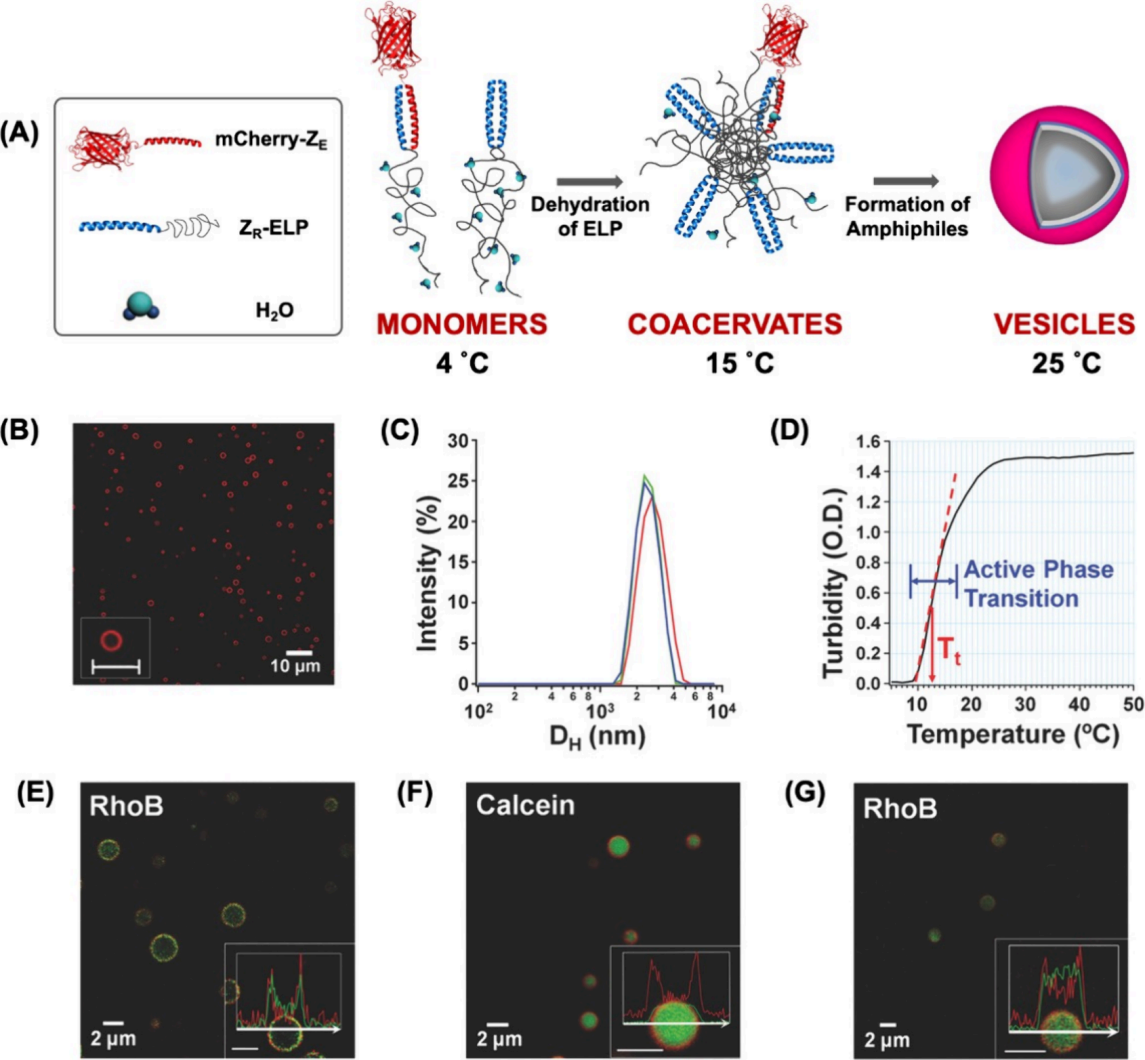
(A) Illustration of the phase transition of mCherry-Z_E_/Z_R_-ELP complexes with increasing temperature. (B) Confocal micrograph (inset scale bar = 5 μm). (C) Hydrodynamic diameter of 3 batches of vesicles. (D) Turbidity (optical density at 400 nm) profile of protein mixture solution (2 mg mL^–1^, 0.05 Z_E_/Z_R_) while increasing the temperature at 1 °C min ^–1^. Confocal micrographs of mCherry-Z_E_/Z_R_-ELP vesicles: (E) double-layered vesicles with hydrophobic dye RhoB (green) trapped within the membrane, (F) double-layered vesicles with hydrophilic dye calcein (green) trapped in the lumen, and (G) single-layered vesicles with RhoB trapped in the lumen. All scale bars are 2 μm. Adapted with permission from ref (1). Copyright 2017 Wiley-VCH GmbH & Co. KGaA, Weinheim.

**Figure 2:**
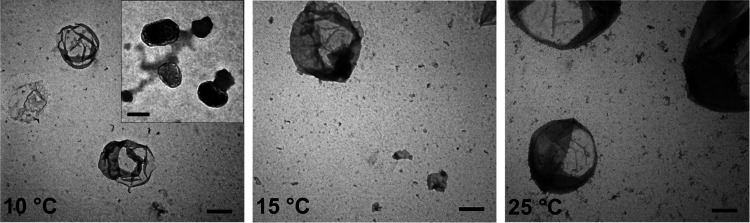
TEM images of self-assembled structures at 10, 15, and 25 °C, made from mCherry-Z_E_/Z_R_-ELP mixture (2 mg mL^–1^, 0.05 Z_E_/Z_R_). Scale bars are 1 μm and 200 nm (inset). Adapted with permission from ref (1). Copyright 2017 Wiley-VCH GmbH & Co. KGaA, Weinheim.

Mixtures of globule-Z_E_ (hydrophilic) and Z_R_-ELP (hydrophobic) form stable heterodimeric complexes via high affinity zipper binding (dissociation constant *K*_D_ ≈ 10^–15^ M).^1,25^ Z_R_ motifs have weaker self-affinity (*K*_D_ ≈ 10^–7^ M)^25^ but form homodimers.^16^ Vesicle self-assembly is driven by the thermal phase transition of the ELP.^1^ In aqueous solution, the globule-Z_E_/Z_R_-ELP complexes and Z_R_-ELP homodimers form by mixing at 4 °C. Then, the protein solution is incubated at room temperature (25 °C) for 1 h (Figure 1A). As the solution warms, the protein complexes become amphiphilic due to the ELP hydrophobic transition, forming monodisperse hollow vesicles with mCherry-Z_E_ homogeneously displayed on the surface (Figure 1B,C). The turbidity of the solution increases due to ELP coacervation.^1^ The turbidity profile during assembly revealed the *T*_t_ of a specific mixture to be ∼12 °C with an active phase transition between 10 and 20 °C that stabilized above 25 °C, suggesting stable vesicle formation (Figure 1D). Self-assembled structures formed at 5, 10, 15, and 25 °C were visualized by transmission electron microscopy (TEM) (Figure 2). No structures were seen at 5 °C. As the phase transition starts around 10 °C, both dense coacervates and hollow vesicles were observed. At 15 and 25 °C, above the *T*_t_, only hollow vesicles were observed, with increased vesicle diameter at higher temperatures. The conformational changes observed throughout vesicle self-assembly are driven by decreased ELP solubility in aqueous solution with coacervates reorganizing into vesicles upon increasing amphiphilicity of the proteins, driving the more soluble mCherry-Z_E_ to the coacervate–water interface to shield the ELP from water. As the temperature increases, the ELP conformation is more collapsed, resulting in reduced vesicle curvature, thereby increasing vesicle size.^1^ Scanning electron microscopy (SEM) imaging of fractured freeze-dried vesicles confirmed that protein vesicles are hollow with an aqueous phase in the center.^16^ Further, small angle neutron scattering (SANS) and dye experiments revealed that vesicles made under most conditions have a single layer with a hydrophobic, ELP-facing lumen but vesicles made at higher protein concentration or higher temperature have a double layer membrane where the ELP domains face each other within the membrane.^1^ SANS analysis indicated that double-layer (bilayer) vesicles exhibited an increase in membrane thickness (22 nm) when compared to single-layer vesicles (13 nm). Further, formation of single- and double-layer vesicles in the presence of hydrophobic and hydrophilic dyes revealed confinement of the hydrophobic dye in the ELP-facing lumen of single-layer vesicles (Figure 1G). Conversely, in double-layer vesicles, the hydrophobic dye was confined within the membrane (Figure 1E) and hydrophilic dye resided in the lumen (Figure 1F).

## 3 Engineering Protein Vesicles

### 3.1 Assembly Conditions

#### 3.1.1 Ionic Strength

Salt concentration is important for vesicle self-assembly because the ELP phase transition depends on ionic strength.^18,22^ Increasing NaCl concentration decreases *T*_t_ and increases ELP hydrophobicity.^22^ Stable vesicle formation is only observed above critical salt concentrations.^16^ Otherwise, unstable coacervates form. At salt concentrations near the minimum, hybrid structures form with a vesicle-like shell and coacervate core.^2^ Each globular protein and the ratio of Z_E_/Z_R_ has a critical salt concentration. mCherry-Z_E_/Z_R_-ELP complexes require less salt than eGFP-Z_E_/Z_R_-ELP because mCherry has a more hydrophilic surface.^26^ This difference suggests that eGFP-Z_E_/Z_R_-ELP complexes are not sufficiently amphiphilic and need more salt to increase the ELP hydrophobicity to form vesicles. We hypothesize that smaller globular proteins need more salt to form vesicles due to higher surface hydrophobicity compared to bigger globular proteins.^2^

**Figure 3:**
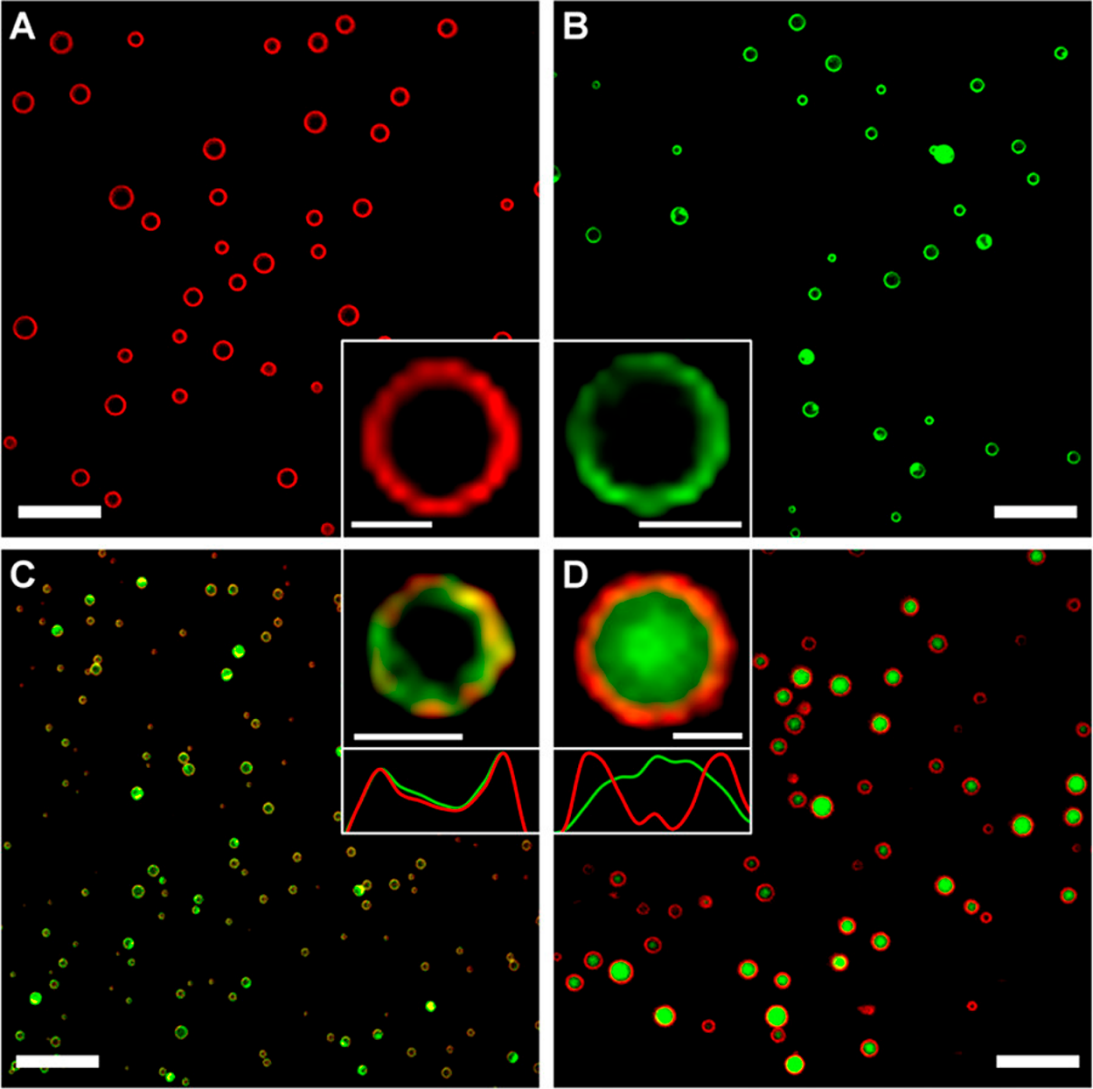
Confocal micrographs of self-assembled protein vesicles prepared from solutions containing different recombinant proteins (Z_R_-ELP concentration is 30 μM for all samples): (A) 1.5 μM mCherry-Z_E_, 0.3 M NaCl (red); (B) 0.6 μM eGFP-Z_E_, 0.91 M NaCl (green); (C) 0.3 μM mCherry-Z_E_, 0.3 μM eGFP-Z_E_, 0.91 M NaCl; (D) 1.5 μM mCherry-Z_E_, 0.6 μM eGFP-Z_E_, 0.45 M NaCl. Large scale bars are 10 μm, and insets are 1 μm. Fluorescence intensity profiles correspond to the insets. Adapted with permission from ref (16). Copyright 2014 American Chemical Society.

We used minimum salt concentration to control vesicle morphology incorporating two globular domains, mCherry-Z_E_ and eGFP-Z_E_. mCherry-Z_E_ and eGFP-Z_E_ vesicles were made separately, each with the required minimum salt concentration (0.3 M, and 0.91 M, respectively) (Figure 3A,B).^16^ In Figure 3C, both globular domains were incorporated homogeneously into the vesicle membrane using 0.91 M NaCl to make yellow vesicles. However, when both globular domains are incorporated at 0.45 M, which favors only mCherry-Z_E_ vesicle formation, mCherry-Z_E_/Z_R_-ELP complexes self-assemble into vesicles that encapsulate eGFP-Z_E_/Z_R_-ELP coacervates (Figure 3D). At this salt concentration, the ELP is not sufficiently hydrophobic to form a balanced amphiphile with eGFP so there is no driving force for reorganization into vesicles.

**Figure 4:**
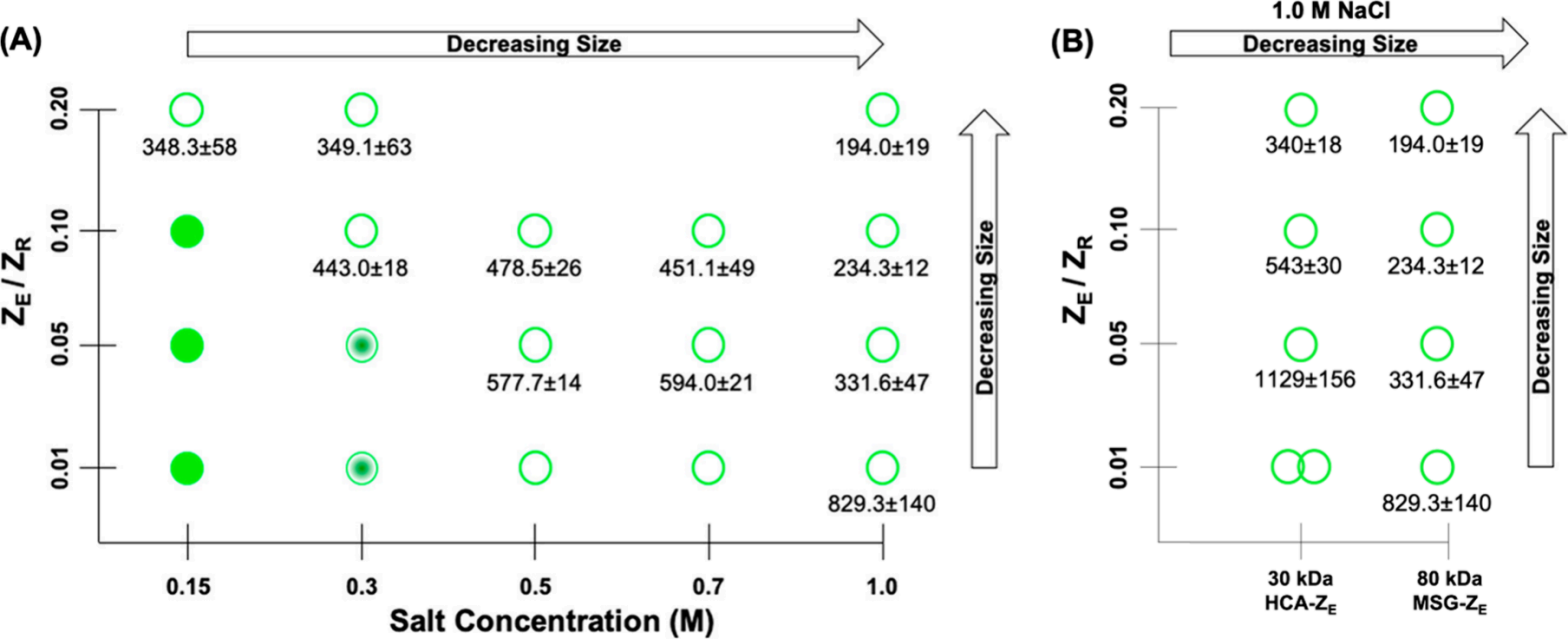
Phase diagrams of 10 μM Z_R_-ELP and (A) MSG-Z_E_ and (B) HCA-Z_E_ and MSG-Z_E_ warmed to 25 °C. Filled circles represent coacervates. Partially filled circles represent the hybrid structures. Hollow circles represent stable vesicles. The hydrodynamic diameters and standard deviations of vesicles in nanometers are listed underneath. Adapted with permission from ref (2). Copyright 2021 American Chemical Society.

Given the effect of salt concentration on vesicles containing different globular proteins, we then explored the effect of ionic strength on vesicle size.^2^ Vesicle diameter decreases with increasing salt concentration (Figure 4A), regardless of the surface charge or globular protein size. Increasing ionic strength increases ELP hydrophobicity, enabling formation of tightly packed smaller vesicles.^2,22^ Additionally, since salt decreases *T*_t_, vesicles spend less time in the coacervate transition phase so there is less time to coalesce and grow before reorganization to stable vesicles.^3^ These results highlight the importance of ionic strength on protein vesicle self-assembly and properties.

#### 3.1.2 Heating Path

**Figure 5:**
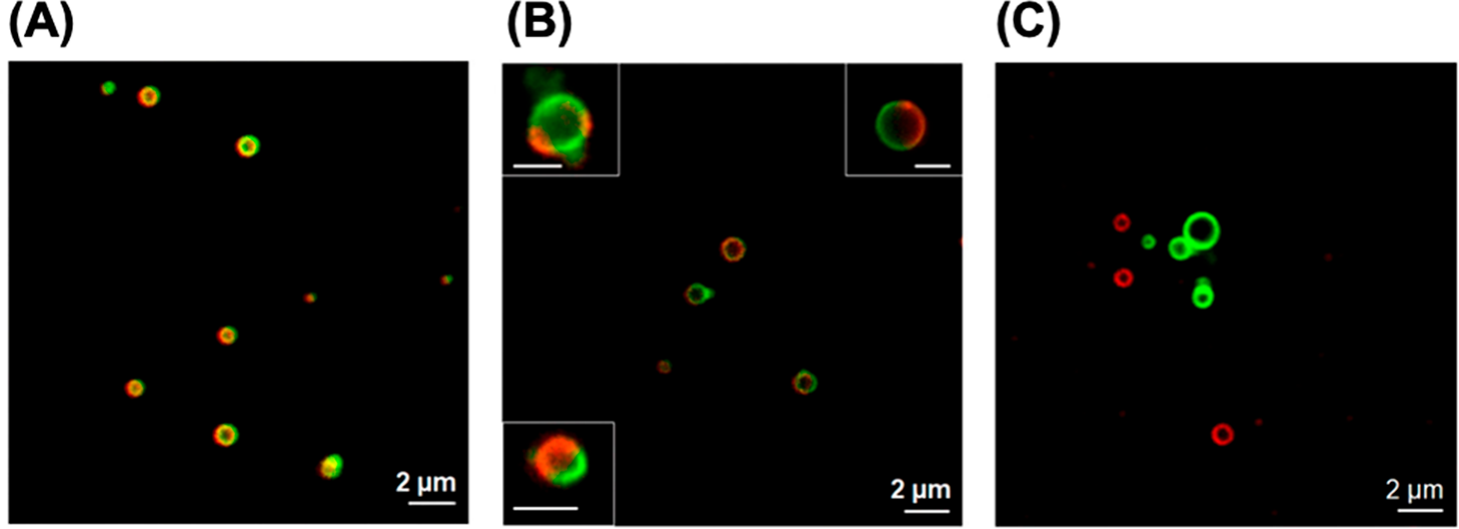
Engineering vesicle membrane morphology by tuning solution incubation pathways: (A) homogeneous mCherry-Z_E_ (red) and eGFP-Z_E_ (green), “yellow” vesicles made from soluble protein mixtures; (B) heterogeneous protein vesicles transitioned from mCherry-Z_E_ and eGFP-Z_E_ coacervate mixtures, and (C) separate mCherry-Z_E_ and eGFP-Z_E_ vesicles mixed after the vesicle transition. Adapted with permission from ref (27). Copyright 2019 American Chemical Society.

The heating path can also affect protein phase transition and vesicle self-assembly.^1,27^ We investigated assembly of mCherry-Z_E_ and Z_R_-ELP at different temperatures (5 °C, 15 °C, and 25 °C) over time. At 10 °C, most of the population was soluble protein, whereas at 25 °C, vesicles formed. At 15 °C, only coacervates were observed, and their diameter increased over time due to coacervates consuming soluble protein or coalescence. Coacervate size directly corresponded to the resulting vesicle size, indicating that the coacervate phase is ideal to manipulate the transition to change vesicle morpohology or size.^27^ We found that the membrane structure of protein vesicles containing two globular domains can be engineered by tuning the time and mixing in the coacervate phase.^27^ This was evident by monitoring the membrane composition of vesicles that contain mCherry-Z_E_, eGFP-Z_E_, and Z_R_-ELP prepared by different pathways. The first pathway consists of mixing protein solutions at 4 °C and warming to 25 °C for 1 h. This resulted in yellow homogeneous vesicles, suggesting that the proteins form homogeneous coacervates and transition into vesicles (Figure 5A). In the second pathway, individual mCherry-Z_E_/Z_R_-ELP and eGFP-Z_E_/Z_R_-ELP solutions were made at 4 °C and incubated at 10 °C for 30 min to form red and green coacervates. Then the coacervates were mixed and held at 10 °C for 30 min to enable coalescence and, finally, warmed to 25 °C for 1 h. This method forms vesicles with heterogeneous membranes displaying each globule-Z_E_ on the surface resulting from limited protein mobility in coacervates (Figure 5B). However, over 4 h the membrane did homogenize, indicating that vesicle membranes are dynamic. The third pathway consists of separately making mCherry-Z_E_/Z_R_-ELP and eGFP-Z_E_/Z_R_-ELP solutions at 4 °C and warming to 25 °C for 1 h to form vesicles. Upon mixing, separate red and green vesicles were maintained, indicating that vesicles are stable and do not coalesce (Figure 5C). This shows that the size and membrane morphology of protein vesicles can be engineered by tuning heating rate, heating path, and mixing.

#### 3.1.3 Protein Concentration and Molar Ratio

Protein vesicle self-assembly can also be tuned by varying the protein concentration and Z_E_/Z_R_ ratio.^1,28^ Increasing the protein concentration decreases *T*_t_.^1,24^ However, this has the opposite effect of a salt-induced decrease of *T*_t_. The average diameter of coacervates and vesicles increased as protein concentration increased. We hypothesize that increasing protein concentration leads to more intermolecular interactions, which translates to increased nucleation sites, resulting in larger coacervates.^29^ With a salt increase, the time spent in the transition decreases, giving less time for coacervate growth and coalescence (Figure 4A).^2^

For constant Z_R_-ELP concentration, increasing the Z_E_/Z_R_ ratio results in smaller vesicles.^2^ While diluting ELP with more globular protein should increase *T*_t_,^30^ the effect is small and the time spent in transition was not significantly altered.^2^ Instead, this behavior is likely caused by increased steric repulsion between globular proteins displayed on the vesicle surface, and curvature increases to compensate.

### 3.2 Genetic Modifications

#### 3.2.1 Globular Protein Modifications

The globule-Z_E_ domain can be varied to impart functionality beyond fluorescence.^2^ We investigated the effect of molecular weight and surface charge of the globular protein on vesicle self-assembly.^2^ The effect of molecular weight was evaluated using three monomeric enzymes fused to Z_E_: human carbonic anhydrase II (HCA, 30 kDa), human glucokinase (HGK, 50 kDa), and *E. coli* malate synthase G (MSG, 80 kDa). We confirmed vesicle self-assembly for each enzyme. Consistent with the effect of increasing the Z_E_/Z_R_ molar ratio, increasing the globular protein size results in smaller vesicles, as seen for HCA-Z_E_ and MSG-Z_E_ vesicles (Figure 4B). This trend was explained similarly; larger proteins have increased steric repulsion and increased curvature is required. Additionally, we investigated the effect of globular protein surface charge by designing monomeric variants of superfolder green fluorescent protein-Z_E_: sfGFP-Z_E_(−10), sfGFP-Z_E_(0), and sfGFP-Z_E_(+10), with net surface charges of −10, 0, and +10, respectively.^2^ All variants formed stable vesicles of similar sizes, suggesting that the charge of the globular protein plays little role in vesicle self-assembly. This was attributed to the high salt concentration used to form the vesicles, screening any electrostatic repulsion between globular proteins.^31^ However, increasing the salt concentration still decreased the size of the vesicles, regardless of the charge of the globular protein.

#### 3.2.2 ELP Modifications

**Figure 6:**
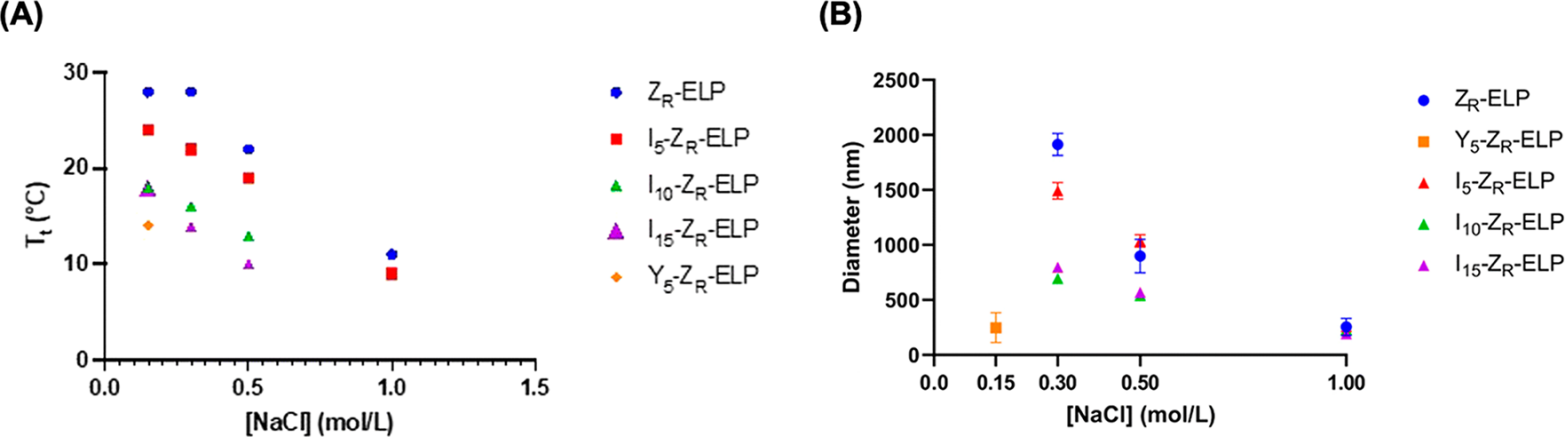
Characterization of (A) *T*_t_ and (B) hydrodynamic diameter of mCherry-Z_E_ (1.5 μM) mixed with I-Z_R_-ELP or Y-Z_R_-ELP variants (30 μM) as a function of NaCl concentration. Error bars represent the standard deviation of the average diameter. Adapted with permission from ref (3). Copyright 2023 Royal Society of Chemistry.

Given the effect that manipulating *T*_t_ through assembly conditions had on vesicle properties, we hypothesized that tuning *T*_t_ through ELP sequence modification could be another route to tune vesicles. We changed the ELP guest residue (X in (VPGXG)_*n*_) and length (*n*) to vary *T*_t_. Urry et al. reported that the hydrophobicity of X inversely influenced *T*_t_, where increasingly hydrophobic amino acids like tyrosine (Y) depressed *T*_t_ compared to less hydrophobic amino acids like valine (V).^23^ Protein engineering enabled analysis of guest residue hydrophobicity influence on vesicle self-assembly by substituting 5, 10, or 15 hydrophobic residues to replace valine (Table 1). Nomenclature starts with the X amino acid abbreviation and uses a subscript for the number substituted. Increasing isoleucine (I) residues at X decreased *T*_t_, leading to decreased protein vesicle size (Figure 6).^3^ As with increasing salt, a decrease in *T*_t_ due to ELP hydrophobicity reduces the time spent in the coacervate phase and, subsequently, vesicle size.^27^ mCherry-Z_E_/I_15_-Z_R_-ELP vesicles had a minimum required salt concentration of 0.3 M NaCl. More hydrophobic tyrosine decreased *T*_t_ further so that nanoscale vesicles formed at 0.15 M NaCl (Figure 6). At higher salt concentrations or with more than 5 tyrosine substitutions, ELP transitioned on ice and only formed hybrid or aggregated nonvesicular structures indicating that too much hydrophobicity leads to imbalanced protein amphiphiles.

**Table 1:** Sequences of the ELP Guest Residue and Length Variants^a^

ELP name	ELP sequence
Z_R_-ELP_25_	[VPGVG VPGVG VPGFG VPGVG VPGVG]_5_
I_5_-Z_R_-ELP	[VPGVG VPG**I**G VPGFG VPGVG VPGVG]_5_
I_10_-Z_R_-ELP	[VPGVG VPG**I**G VPGFG VPG**I**G VPGVG]_5_
I_15_-Z_R_-ELP	[VPG**I**G VPG**I**G VPGFG VPG**I**G VPGVG]_5_
Y_5_-Z_R_-ELP	[VPGVG VPG**Y**G VPGFG VPGVG VPGVG]_5_
Z_R_-ELP_15_	[VPGVG VPGVG VPGFG VPGVG VPGVG]_**3**_
Z_R_-ELP_35_	[VPGVG VPGVG VPGFG VPGVG VPGVG]_**7**_
Z_R_-ELP_50_	[VPGVG VPGVG VPGFG VPGVG VPGVG]_**10**_
H_5_-Z_R_-ELP	[VPGVG VPG**H**G VPGFG VPGVG VPGVG]_5_
H_10_-Z_R_-ELP	[VPGVG VPG**H**G VPGFG VPG**H**G VPGVG]_5_
H_15_-Z_R_-ELP	[VPG**H**G VPG**H**G VPGFG VPG**H**G VPGVG]_5_
pZ_R_-ELP	[VPGVG VPGVG VPGpAzFG VPGVG VPGVG]_5_

a Phenylalanine residues (F) within pZ_R_-ELP are partially replaced by *para*-azido-phenylalanine residues (pAzF) during protein expression.

**Figure 7:**
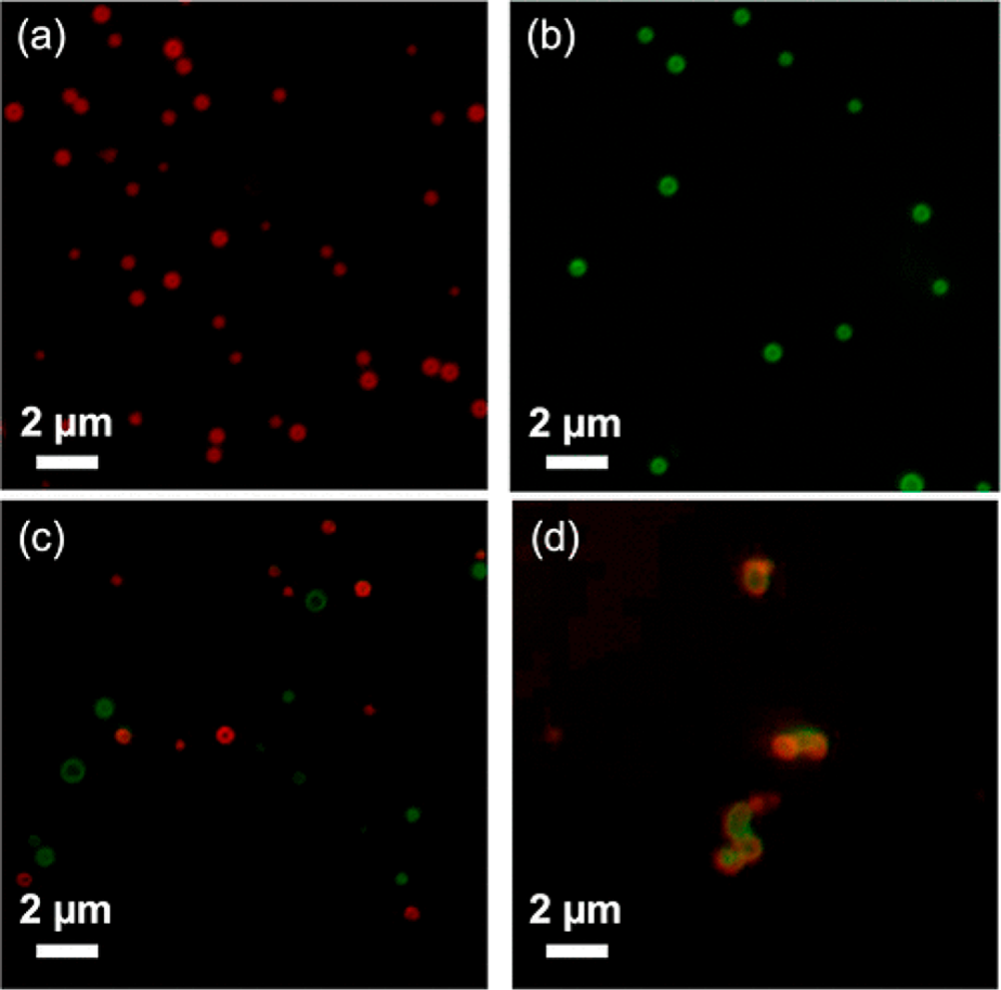
pH responsive vesicle fusion at a low pH. (A) mCherry-Z_E_/H_5_-Z_R_-ELP vesicles at pH 7.5. (B) sfGFP-Z_E_/H_5_-Z_R_-ELP vesicles at pH 7.5. (C) Mixture of mCherry-Z_E_/H_5_-Z_R_-ELP and sfGFP-Z_E_/H_5_-Z_R_-ELP vesicles at pH 7.5. (D) Mixture of mCherry-Z_E_/H_5_-Z_R_-ELP and sfGFP-Z_E_/H_5_-Z_R_-ELP vesicles at pH 6.5 for 1 h. Adapted with permission from ref (32). Copyright 2022 American Chemical Society.

Given the effect that manipulating *T*_t_ through guest residue hydrophobicity had on vesicle properties, we hypothesized that including an ionizable amino acid, histidine (H, p*K*_a_ = 6.0), in the ELP could impart pH-responsiveness. Incorporating histidine into ELP causes intra- and intermolecular electrostatic repulsion between charged groups at acidic pH, forcing ELP to adopt a more extended hydrophilic conformation.^32^ The difference in *T*_t_ of mCherry-Z_E_/H-Z_R_-ELP protein solutions between pH 8 and 5.5 was measured. Greater histidine content increased pH responsiveness, analogous to the greater change in *T*_t_ with an increasing number of hydrophobic guests. *T*_t_ increased with decreasing pH and with increasing histidine content (5, 10, 15, Table 1). Vesicles made at mildly acidic pH with protonated histidine were larger than those formed at neutral or basic pH, analogous to larger vesicles forming from higher *T*_t_ ELPs with less hydrophobic substitutions than more hydrophobic ELPs with lower *T*_t_ values. This trend was observed at 0.5 M NaCl, indicating that charge screening did not seem to interfere with the behavior of charged ELPs. Additionally, vesicles containing the histidine ELP formed at basic pH grew larger and completely disassembled over time at acidic pH as the ELP hydrophobic transition reversed back to hydrophilic. Using fluorescent red and green vesicles, we elucidated that vesicles at acidic pH fused (rather than swelled) prior to disassembly into soluble proteins (Figure 7). Vesicles likely fused due to instability caused by increased hydrophilicity of the ELP, perhaps similar to behavior of liposomes undergoing hexagonal phase driven membrane fusion.^33^

Since guest residue substitutions influence vesicle assembly and disassembly, we hypothesized that changing ELP length (*n* = 15, 25, 35, 50; Table 1) would also impact assembly.^3^ As the ELP length increases, *T*_t_ decreases, resulting in smaller vesicles, also due to less time spent in the coacervation stage. Z_R_-ELP_50_ decreased the *T*_t_ sufficiently that the required salt for vesicle assembly was reduced from 0.3 to 0.15 M. Z_R_-ELP_50_ and Y_5_-Z_R_-ELP were the only sequences capable of inducing vesicle assembly at a low (physiological) 0.15 M salt. Comparing these two genetic variants at 0.15 M salt revealed that the addition of 5 tyrosines to the ELP guest residues had a much more significant effect on reducing vesicle size than doubling the ELP length from 25 to 50 as the tyrosine vesicles were over 5× smaller than vesicles made with 50 ELP repeats. Even when the salt was increased to 1.0 M NaCl, vesicles formed from the longest ELP were still over twice as large as tyrosine vesicles made at low salt (the only concentration possible for tyrosine vesicles). Although the longer ELP has a slightly lower *T*_t_, longer ELP chains cannot pack as tightly as shorter chains. Therefore, increasing ELP hydrophobicity was more effective in reducing vesicle size for drug delivery applications and enabled assembly at physiological salt. However, longer ELPs could reduce membrane dynamics and diffusion resulting in prolonged membrane heterogeneity, which could be useful for targeting or vaccine applications.

**Figure 8:**
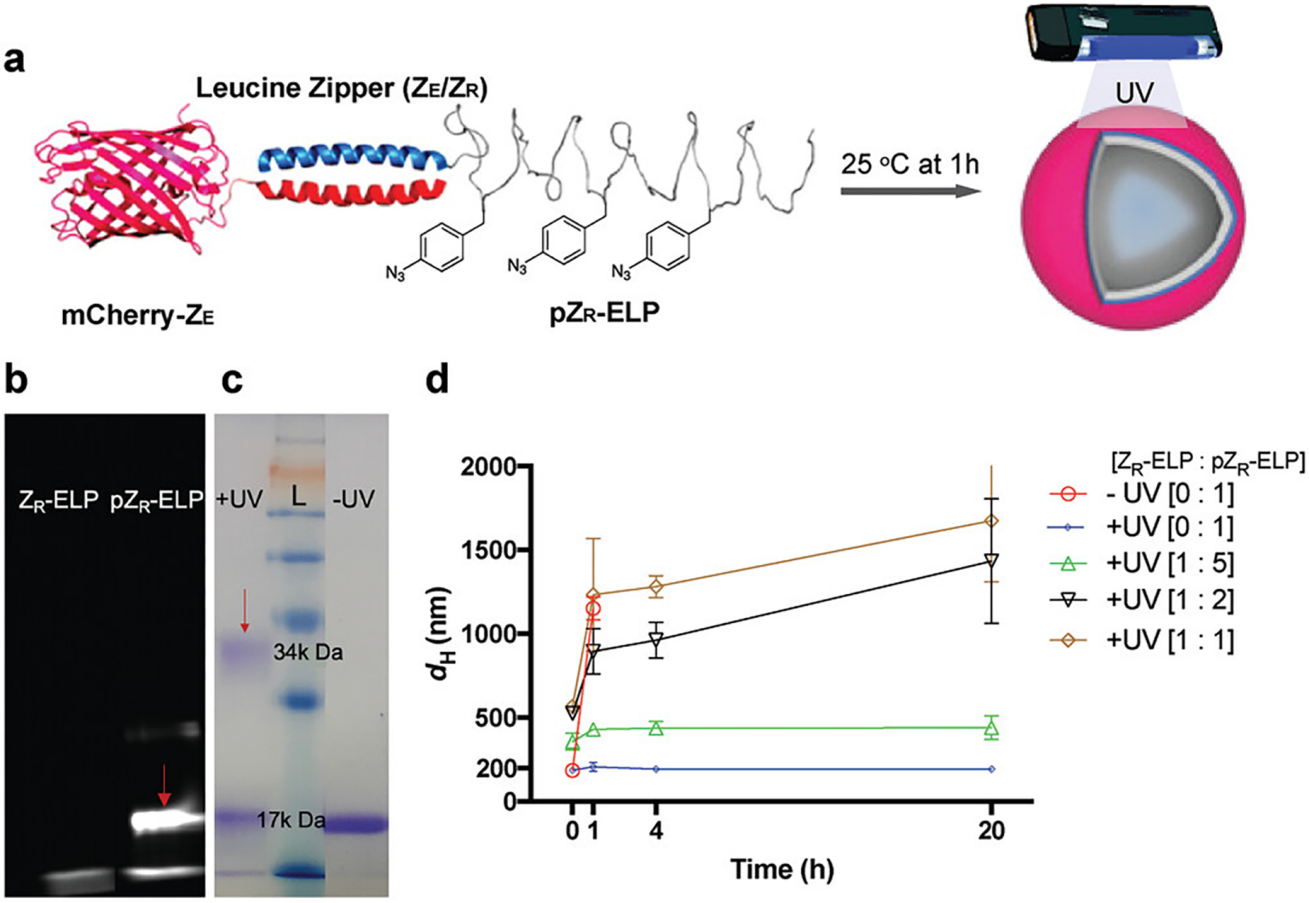
Incorporation of pAzF into Z_R_-ELP and photocrosslinking of mCherry-Z_E_/pZR-ELP vesicles. a) Schematic of thermally triggered self-assembly and photocrosslinking of mCherry-Z_E_/pZ_R_-ELP vesicles. Phenylalanine guest residues in Z_R_-ELP were partially replaced by pAzF. b) Fluorescent SDSPAGE gel of Z_R_-ELP and pZ_R_-ELP containing pAzF residues reacted with dibenzocyclooctyne (DBCO)-conjugated Cy5 via a click reaction showed fluorescence only for pZ_R_-ELP at 17 kDa (red arrow). c) SDS-PAGE gel of pZ_R_-ELP with and without UV irradiation shows higher molecular weight species formed by UV crosslinking (red arrow). L is the ladder. d) Hydrodynamic diameters of protein vesicles with and without UV irradiation. Vesicles made from Z_R_-ELP and pZ_R_-ELP mixed at different ratios (0:1, 1:5, 1:2, and 1:1) displayed different sizes and degrees of swelling and were stable at least 20 h, while vesicles without UV irradiation disassemble within 4 h. Data represent the mean ± standard deviation (*n* = 3). Adapted with permission from ref (34). Copyright 2021 Wiley-VCH GmbH & Co. KGaA, Weinheim.

While vesicle disassembly is desired in some applications, we also sought to modify the ELP sequence to make vesicles stable in a variety of conditions by cross-linking. We used a photoreactive non-natural amino acid, *para*-azido-phenylalanine (pAzF), to cross-link ELP using UV irradiation (Figure 8).^34^ Z_R_-ELP was coexpressed with a mutant phenylalanyl-tRNA synthetase in the phenylalanine auxotroph *E. coli* strain, AFIQ, with pAzF replacing phenylalanine in the media to create pZ_R_-ELP (Table 1).^35,36^ Vesicles were formed at high NaCl concentration and underwent UV irradiation induced cross-linking. When diluted in physiological salt (0.15 M), they retained their structure for at least 40 days at room temperature. Without UV irradiation, vesicles disassembled within 4 h upon dilution. pZ_R_-ELP containing vesicles were also ∼4-fold smaller than Z_R_-ELP vesicles because pZ_R_-ELP is more hydrophobic than Z_R_-ELP. The ratio of Z_R_-ELP to pZ_R_-ELP used in vesicle assembly dictated the diameter and degree of swelling upon dilution of salt from 1.0 M to 0.15 M (Figure 8).

Altogether there are many routes, via changing assembly conditions or genetic sequence, to tune protein vesicle size, structure, stimuli-responsiveness, or stability. Varying assembly conditions is the most cost-effective and timely way to change vesicle characteristics. However, vesicles are thermodynamically stable only under the conditions of their assembly. Modifying the ELP sequence gives control over stability and disassembly under desired conditions, in addition to size. Genetic synthesis or mutation requires more time and resources up front than changing assembly conditions, but the proteins can be used in different vesicle formulations with no additional effort.

## 4 Applications of Protein Vesicles

The characteristics of self-assembled protein vesicles can be tuned by either genetic modifications or changes in the assembly conditions depending on the intended use. A unique aspect of these vesicles is that they are made from folded, functional, globular proteins with a preserved structure and functionality. Vesicles can be made from a wide variety of globular proteins with molecular weights and surface charges ranging from at least 27 to 80 kDa and from −10 to +10, respectively. We have explored a variety of applications for protein vesicles including biocatalysis, vaccines, and drug delivery.

### 4.1 Biocatalysis

Enzyme immobilization can improve the effectiveness of biocatalysts and impart reusability.^37^ Self-assembling proteins have gained interest as enzyme immobilization platforms due to their tunability.^38^ Self-assembling protein vesicles are a promising platform for enzyme immobilization, as vesicles are stable and tunable and preserve the functionality of the fusion proteins. We investigated self-assembly of vesicles using monomeric enzymes fused to Z_E_.^2^ Initial enzyme activity was measured for soluble Z_E_ fused enzymes and vesicles made from enzymes (MSG-Z_E_, HGK-Z_E_, and HCA-Z_E_). MSG-Z_E_ exhibited the same activity in both soluble and vesicle form, though vesicles still provide the benefit of enzyme recovery and reuse, extending their lifetime and reducing cost. Conversely, HGK-Z_E_ vesicles exhibited ∼40% decrease in activity. This was attributed to the enzyme remaining in small coacervate-like structures inside the vesicles, likely due to four unpaired cysteines in HGK that formed interprotein disulfide bonds during coacervation. This likely led to aggregation within the coacervate and prevented complete reorganization. It also identifies the protein structure as important in dictating whether a protein can form functional vesicles. Alternately, esterase activity of HCA-Z_E_ vesicles exhibited a 6.5-fold increase when immobilized. We hypothesize that this increase could be caused by localization of the hydrophobic substrate to the ELP inside vesicles, providing an adjacent source of substrate that increases the local concentration near the enzyme. Both MSG and HGK substrates are soluble, while the HCA substrate has poor aqueous solubility. This observation opens the potential for vesicles to provide an advantage to enzymes with insoluble substrates, which could eliminate the need for two phase aqueous–organic solvent biocatalysis. Altogether, these results demonstrate the robustness of protein vesicles and their potential for biocatalytic applications with enzymes.

### 4.2 Vaccines

The ability of protein vesicles to display proteins on the surface motivated investigation of vesicles as vaccines.^39^ Self-assembling vaccine platforms are beneficial because they display (often peptide) antigens on the surface, preserve antigen structure, and maintain orientation.^40^ We investigated the efficacy of protein vesicles to deliver a full size antigen protein and evaluated *in vivo* immune responses.^39^ This was achieved using model antigen ovalbumin (OVA) fused to Z_E_ and self-assembled with pZ_R_-ELP to form cross-linked nanoscale vesicles (Figure 8A). We immunized mice and assessed the antibody and T cell responses. After prime immunization with the OVA vesicles, the OVA-specific IgG1 and IgG2a antibody responses were apparent, while there was no detectable response in the soluble group. After boost, OVA vesicles increased IgG1 titer approximately 20-fold higher than soluble OVA-Z_E_. No IgG2a was observed for soluble OVA-Z_E_, but IgG2a titers increased after the OVA vesicle boost. IgG1 and IgG2a are two subclasses of immunoglobulin. Due to structural differences, they elicit different effector functions to protect against pathogens.^41^ Splenic T cells were also collected, and we determined that OVA vesicles induced more OVA-specific CD4^+^ (helper) T cells producing IL-4 and more CD8^+^ (cytotoxic) T cells producing both IL-4 and IFN-γ compared to soluble OVA-Z_E_. Type 1 T helper cells secrete IL-4, which induces B cell class switching to produce IgG1 but inhibits IgG2a production. Type 2 helper cells secrete IFN-γ which induces B cell class switching to produce IgG2a and inhibits IgG1 production.^42^ This work suggests that protein vesicles are a beneficial subunit vaccine platform for enhanced immune responses because of their ability to present full size antigens and stability, while the ability to vary the size and antigen density could be valuable in future studies.

### 4.3 Drug Delivery

ELP materials are biocompatible and micelles^43^ and vesicles^13^ containing ELP have been used for drug delivery. Drug delivery requires efficient drug loading and, for intracellular delivery, requires endosomal escape with cargo release for drugs with sites of action in the cytosol. Small, hydrophobic drugs can enter cells directly, while larger therapeutics are poorly delivered without a carrier. For drug delivery applications, the optimal carrier size is 10–200 nm in diameter, as smaller or larger sizes result in rapid clearance.^44,45^ To use vesicles for delivery applications, we engineered vesicles with ideal size, stability, and release capabilities for small molecule and protein cargos. We formulated stable, photo-cross-linked protein vesicles for delivery of small molecule doxorubicin (Dox) (Figure 8).^34^ Dox was encapsulated by mixing with pZ_R_-ELP and mCherry-Z_E_ protein solutions on ice and warming above the *T*_t_. Vesicles formed using 2 M NaCl had an encapsulation efficiency >80%, indicating that vesicles capture Dox during self-assembly due to attractive hydrophobic interactions between Dox and ELP.^16^ When dialyzed into physiological salt concentration, the ELP transition reversed to hydrophilic, reducing the hydrophobic attraction and releasing Dox over 30 h despite the cross-linked vesicles maintaining their shape. Dox loaded vesicles were internalized by HeLa cells and induced cytotoxicity, indicating their successful release inside cells. While Dox does not need a carrier to enter cells, in future work mCherry-Z_E_ can be replaced with a targeting protein so that Dox, or other small, hydrophobic cargo are only delivered to targeted, diseased cells.

To deliver larger hydrophilic molecules that are unable to enter cells and are too big to diffuse through the cross-linked vesicle membrane, a new mechanism was utilized to encapsulate cargo and induce vesicle disassembly within the endosome to yield cargo release. For demonstration, we delivered protein cargos, which are poorly delivered into the cytosol without a carrier.^46^ A mixture of tyrosine and histidine modified Z_R_-ELPs was selected to form vesicles that are both stable in physiological salt concentration and capable of pH triggered disassembly. During endocytosis, there is a change from pH 7.4 to pH 5–6 depending on the endosome stage, which can serve as a trigger for vesicle disassembly and cargo release. Additionally, to enable protein cargo release and cytosolic delivery, we utilized hydrophobic ion pairing (HIP), which temporarily increases the hydrophobicity of a hydrophilic cargo by mixing with an oppositely charged hydrophobic counterion.^46^ HIP loaded vesicles enabled the effective cytosolic delivery of cytochrome c cargo, a protein that induces caspase-mediated cell death only when delivered into the cytosol. HIP cytochrome c loaded vesicles resulted in less than 5% cell viability in HeLa cells after 48 h, while vesicles loaded with cytochrome c without HIP showed no effect on viability. Similar results were seen for an organoid model hosting K562 acute myeloid leukemia cells, demonstrating not only that vesicles delivered and released protein cargo, but the necessity for HIP cargo loading. These protein and small molecule drug delivery results show the potential for protein vesicles for delivering cargos of different size and hydrophobicity for a variety of applications and point to future capabilities for multicargo delivery.

## 5 Summary and Future Outlook

Self-assembled recombinant protein vesicles are biodegradable and biocompatible carriers with controllable physicochemical properties that are most importantly made from large, folded functional proteins. The functional protein building blocks can be altered to obtain the desired functionality and properties of protein vesicles. In this Account, we discussed fundamentals of protein vesicles, our work focused on engineering protein vesicles, and their diverse applications in the fields of biocatalysis, vaccines, and drug delivery. We can engineer the size and membrane morphology of protein vesicles by tuning the heating and mixing steps, resulting in vesicles with homogeneous or heterogeneous membranes as well as single or double layered vesicles. The size of protein vesicles can be controlled by tuning the ionic strength, protein concentration, Z_E_/Z_R_ ratio, and ELP sequence. The choice of globular protein (globule-Z_E_) imparts specific functionality to vesicles and impacts the vesicle size. Future work will reveal if vesicles can accommodate globular proteins outside the 27–80 kDa size range, though unpublished data suggests that 16 kDa may be too small to achieve sufficient amphiphilicity. With vesicles made from 9 distinct globular proteins, and counting, our protein vesicles have use in a variety of applications. Self-assembled enzymatic vesicles retained or improved enzyme activity, highlighting their potential for biocatalysis applications especially in cases where activity could benefit from ELP partitioning of hydrophobic substrates. Moreover, the OVA-Z_E_ vaccine vesicles elicited enhanced antibody and cellular immune responses compared to soluble OVA. This platform is now ripe to adapt to other antigens and mixtures of antigens to create novel subunit vaccine formulations. Finally, we reported intracellular, cytosolic delivery of protein cargo using pH-responsive vesicles and hydrophobic ion pairing. Next steps will reveal if other biomacromolecule cargos can be delivered and the potential for targeting by replacing mCherry with different targeting proteins. Our work on protein vesicles has provided a practical and fundamental understanding of their self-assembly, tunability, and potential for diverse applications. Given the various applications of protein vesicles, a further understanding of vesicle interactions with biological systems *in vitro* and *in vivo* is still necessary to further understand the role of modified ELPs and vesicle size for delivery applications. For example, how do vesicles escape the endosomal pathway and what is the fate of the ELP proteins are *in vitro* questions that we have. *In vivo*, we will need to determine biodistribution and lifetime following administration by different systemic and local routes to identify the most appropriate applications. Overall, given the characteristics of protein vesicles, their applications can be further expanded into new fields, such as artificial cell platforms, biosensors, and microreactors.^47,48^

## Abbreviations

Z_E_: glutamate-rich leucine zipper
Z_R_: arginine-rich leucine zipper
ELP: elastin-like polypeptide
pAzF: *para*-azido-phenylalanine
HCA: human carbonic anhydrase II
HGK: human glucokinase
MSG: *E. coli* malate synthase G
HIP: hydrophobic ion pairing
